# Short-Wavelength (Violet) Light Protects Mice From Myopia Through Cone Signaling

**DOI:** 10.1167/iovs.61.2.13

**Published:** 2020-02-12

**Authors:** Ryan Strickland, Erica G. Landis, Machelle T. Pardue

**Affiliations:** 1 Neuroscience, Emory University, Atlanta, Georgia, United States; 2 Atlanta Veterans Affairs Healthcare System, Atlanta, Georgia, United States; 3 Biomedical Engineering, Georgia Institute of Technology, Atlanta, Georgia, United States

**Keywords:** violet light, cones, myopia

## Abstract

**Purpose:**

Exposure to short-wavelength light influences refractive development and inhibits myopic development in many animal models. Retinal mechanisms underlying this response remain unknown. This study used a mouse model of lens-induced myopia to evaluate the effect of different wavelength light on refractive development and dopamine levels in the retina. A possible retinal pathway is tested using a mutant mouse with dysfunctional cones.

**Methods:**

Wild-type C57BL/6J (WT) and ALS/LtJ/Gnat2^cpfl3^ (*Gnat2^−^^/^*^−^) mice were exposed to one of three different light conditions beginning at postnatal day 28: broad-spectrum “white” (420-680 nm), medium wavelength “green” (525 ± 40 nm), and short wavelength “violet” (400 ± 20 nm). One-half of the mice received hyperopic lens defocus. All mice were exposed to the light for 4 weeks; animals were measured weekly for refractive error and axial parameters. Retinal dopamine and the dopamine metabolite 3,4-dihydroxyphenylacetic acid were measured by HPLC.

**Results:**

In WT mice, short-wavelength violet light induced hyperopia and violet light inhibited lens-induced myopia when compared with mice exposed to white light. Hyperopia could be attributed to shallower vitreous chambers in WT animals. There were no changes in the levels of dopamine or its metabolite. In *Gnat2^−^^/^*^−^ mice, violet light did not induce hyperopia or inhibit lens-induced myopia.

**Conclusions:**

These findings show that short-wavelength light slows refractive eye growth, producing hyperopic responses in mice and inhibiting lens-induced myopia. The lack of inhibition in mice with dysfunctional cones suggests that cone signaling plays a role in the hyperopic response to short-wavelength (violet) light.

Myopia, one of the most prominent visual disorders in the world, is characterized by a mismatch between the optical power of the eye and its axial length, such that light is focused in front of the retina.^1^ Experimental evidence suggests that early visual experiences influence the development of myopia.^2^ For instance, bright, outdoor light exposure in children can be protective against myopia.^3^^–^^5^ Similarly, high ambient lighting has slowed the progression of myopia in animal models of myopia.^6^

While outdoor sunlight has higher luminance levels, it also has a different spectral composition when compared to typical indoor lighting; outdoor sunlight has more intense short-wavelength radiation.^7^^,^^8^ Monochromatic wavelengths have been of interest in refractive eye growth because of the known aberrations created by the eye in response to different wavelengths.^9^^–^^11^ Specifically, the longitudinal chromatic aberrations (LCA) created by the eye result in wavelength-dependent focal planes, such that long wavelengths focus behind the retina and short wavelengths focus in front of the retina. Evidence suggests that the difference in focal planes is significant enough to affect accommodation and emmetropization.^12^^–^^15^ However, the mechanisms by which LCA affects emmetropization are still unknown.

Experimentally, animal models exposed to monochromatic light show variable results. Rhesus monkeys^16^^,^^17^ and tree shrews^18^ developed hyperopic responses when exposed to long-wavelength red light. In contrast, chickens^13^^,^^19^^,^^20^, fish^21^^,^^22^, and guinea pigs^23^^–^^26^ became hyperopic with short-wavelength light exposure. Furthermore, chicks^27^ and guinea pigs^28^ were protected against lens-induced myopia (LIM) when exposed to short wavelength violet and blue light, respectively. Elevated levels of dopamine (DA) in the retina are related to the inhibition of myopic growth during refractive development.^29^^,^^30^ Recent evidence in chickens suggests that DA levels increase in response to short-wavelength light, even when form deprivation is applied.^31^ These results suggest that wavelength cues are influential on refractive development and that short-wavelength light may be protective against myopic growth through increased dopamine levels.

Determining how the mammalian eye responds to wavelength cues is important to understanding retinal mechanisms that may be similar to that of the human eye. Of the animal models examined, guinea pigs are the only mammal that have shown protection against LIM with short-wavelength light.^28^ Another mammalian model of myopia, the mouse, has not been characterized under similar monochromatic conditions. Guinea pigs and mice are dichromatic, but unlike the guinea pig with a maximum sensitivity between 429 and 529 nm^32^, the mouse retina contains both UV- and medium wavelength-sensitive cones (365 and 508 nm peak sensitivity, respectively).^33^^–^^35^ Despite differences in spectral sensitivity, the structure and signaling pathways of the mouse retina closely resemble the human retina. A study using wavefront sensing to characterize the chromatic aberrations created by the mouse eye estimated that roughly 10 diopters (D) of chromatic aberrations exist between 360 and 600 nm light.^36^ The magnitude of this difference in the mouse eye suggests that refractive growth would be affected by monochromatic light exposure. The mouse also offers the opportunity to probe certain signaling pathways through genetic manipulation, which can provide evidence for the involvement of mechanisms that may underlie the mammalian response to LCA. In this study, we examined the refractive development and effect of lens defocus on wild-type (WT) mice after exposure to monochromatic lighting. To examine the potential underlying mechanisms, we measured retinal DA and tested the contribution of retinal cone pathways by examining mice that lacked functional cones.

## Methods

### Animals and Housing

The animals used in this study were age-matched male and female WT C57BL/6J mice (Jackson Laboratory, Bar Harbor, ME) (n = 44) and ALS/LtJ/Gnat2^cpfl3^ (*Gnat2^−^^/^*^−^) mice (Jackson Laboratory) on a C57BL/6J background (n = 49). An in-house breeding colony of each strain was maintained at the Atlanta Department of Veterans Affairs Healthcare System. *Gnat2^−^^/^*^−^ mice have a missense mutation that affects the α-subunit of transducin in cone photoreceptors, effectively preventing the phototransduction cascade within cones.^37^ The dysfunctional cone responses to light have been demonstrated by poor photopic ERG responses by three weeks of age.^37^^,^^38^

**Figure 1.**
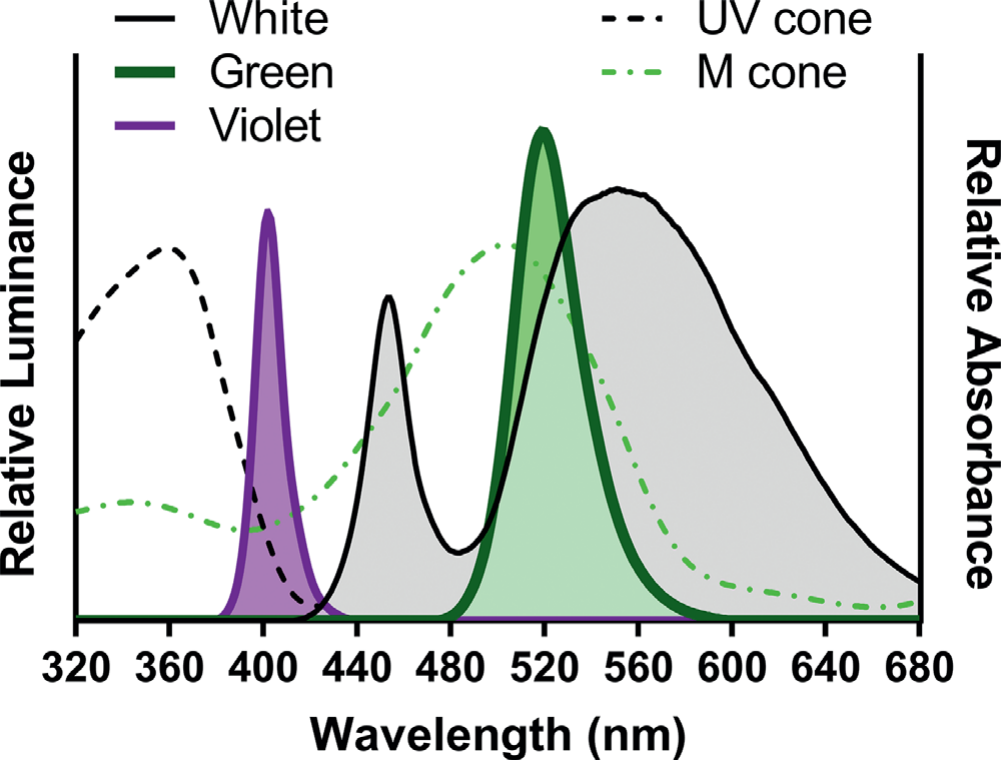
Wavelength spectrums and cone pigment absorbance. Relative wavelength spectrums for the monochromatic ambient light in which animals were housed and photopigment absorbance of the mouse UV and medium-wavelength sensitive cone photoreceptors (dotted lines). Cone absorbance based on Jacobs and Williams.^40^

At postnatal day 28 (P28), mice were transferred in a random fashion from standard fluorescent lighting to one of three custom, ventilated light boxes with light-emitting diodes (LEDs) of different wavelengths. All lights were on a 12:12 hour light:dark cycle. The three different LED light conditions used were: broad-spectrum “white” (420-680 nm), medium-wavelength “green” (525 ± 40 nm), and short-wavelength “violet” (400 ± 20 nm) (NFLS-X3-LC2; Super Bright LEDs Inc., St. Louis, MO) (Fig. 1). The treatment group names of these wavelengths do not indicate that these are the colors that the mice perceive. Light intensity was standardized across the different LEDs to approximately 50 candela/m^2^ from the bottom of the cage as measured with an Exemplar Smart CCD Spectrometer (B&W Tek, Newark, DE). Wavelengths were chosen based on the photosensitivity of the mouse retina which peaks at 365 and 508 nm (Fig. 1),^38^^–^^40^ and the selected light intensity likely involved both rod and cone input.^6^^,^^41^ Cages had wire tops with water and standard mouse chow ad libitum; food was placed on the cage floor to prevent excess shadows. Animal well-being was checked daily. All procedures were approved by the Atlanta Veterans Affairs Institutional Animal Care and Use Committee and conformed to the ARVO Statement for the Use of Animals in Ophthalmic and Vision Research.

### Refractive Development and Lens Defocus

At P28, before monochromatic light exposure, animals underwent baseline measurements for refractive error and ocular biometry, as previously described.^42^^–^^44^ In a dark room, eyes were dilated with 1% tropicamide and animals were anesthetized with ketamine (80 mg/kg) and xylazine (16 mg/kg). Refractive error was measured using a custom-made, automated photorefractor.^45^ Animals were not used if the difference in refraction between their two eyes was greater than 2.5 D (<5% of mice tested at baseline).

Immediately after refractions, the corneal radius of curvature was measured using an automated and customized keratometer.^46^^,^^47^ Subsequently, axial parameters were obtained using a Bioptigen Envisu 4300 System (SD-OCT; Leica, Buffalo Grove, IL).^48^

Following all baseline ocular measurements, a subset of mice underwent surgery (WT n = 17, *Gnat2^−^^/^*^−^ n = 21)^48^^,^^49^ to fasten a head pedestal to the skull and subject the right eye (OD) to hyperopic lens defocus using a transparent, -10 D lens (X-Cel Specialty Contact, Duluth, GA). To reverse the effects of anesthesia, all mice received atipamezole (1 mg/kg) (Antisedian; Zoetis Services LLC, Parsippany, NJ). Sterile saline eye drops were applied to prevent corneal dehydration and animals were placed on a heating pad for recovery. Compliance with lens placement and ocular health was monitored daily; animals with poor compliance (n = 4) or ocular health (n = 3) were excluded from further analysis, which resulted in loss of data at later timepoints.^44^

### Retinal Dopamine Turnover

Animals were sacrificed via cervical dislocation approximately 48 hours after the final ocular measurements were taken to eliminate any effect of anesthesia. Levels of retinal DA and 3,4-Dihydroxyphenylacetic acid (DOPAC), a metabolite of DA,^50^ were determined by collecting retinas between 4 to 6 hours after light onset on the day of sacrifice. Retinas were extracted under dim red light, immediately frozen on dry ice, and stored at -80°C. The retinas were then processed as previously described.^51^ Briefly, 0.1M perchloric acid was added to homogenized samples which were then filtered to remove debris. Supernatant was used to detect monoamine content with HPLC. For HPLC, an ESA 5600A CoulArray detection system was used. Separations were performed at 28 to 30°C using an MD-150 × 3.2 mm C18 column. The mobile phase consisted of 1.4 to 1.7 mM 1-octanesulfonic acid sodium, 75 mM NaH_2_PO_4_, 0.025% triethylamine, and 8% acetonitrile at pH 2.93-3.0. 20 µL of sample was injected. The analytes were identified by matching the retention time to known standards (Sigma Chemical Co., St. Louis, MO). Compounds were quantified by comparing peak areas with those of standards.

### Statistical Analyses

The right eye of mice that were not lens-treated and both eyes of the mice with lens defocus (lens-treated and contralateral) were analyzed here. Data collected with SD-OCT was obtained by using a custom-made MATLAB program (The MathWorks, Inc., Natick, MA) to measure ocular structures offline. Longitudinal data obtained from the photorefractor, keratometer, and SD-OCT were analyzed with repeated measures two-way ANOVAs (SigmaPlot, Systat Software, Inc., Chicago, IL). Data from a single timepoint was analyzed using either one- or two-way ANOVAs. For all calculations, Holm-Sidak *post-hoc* procedures were used to make pairwise comparisons. All data are represented as the mean ± SEM.

## Results

### Short-Wavelength (Violet) Light Exposure Induced Hyperopic Refractions in WT Mice

**Figure 2.**
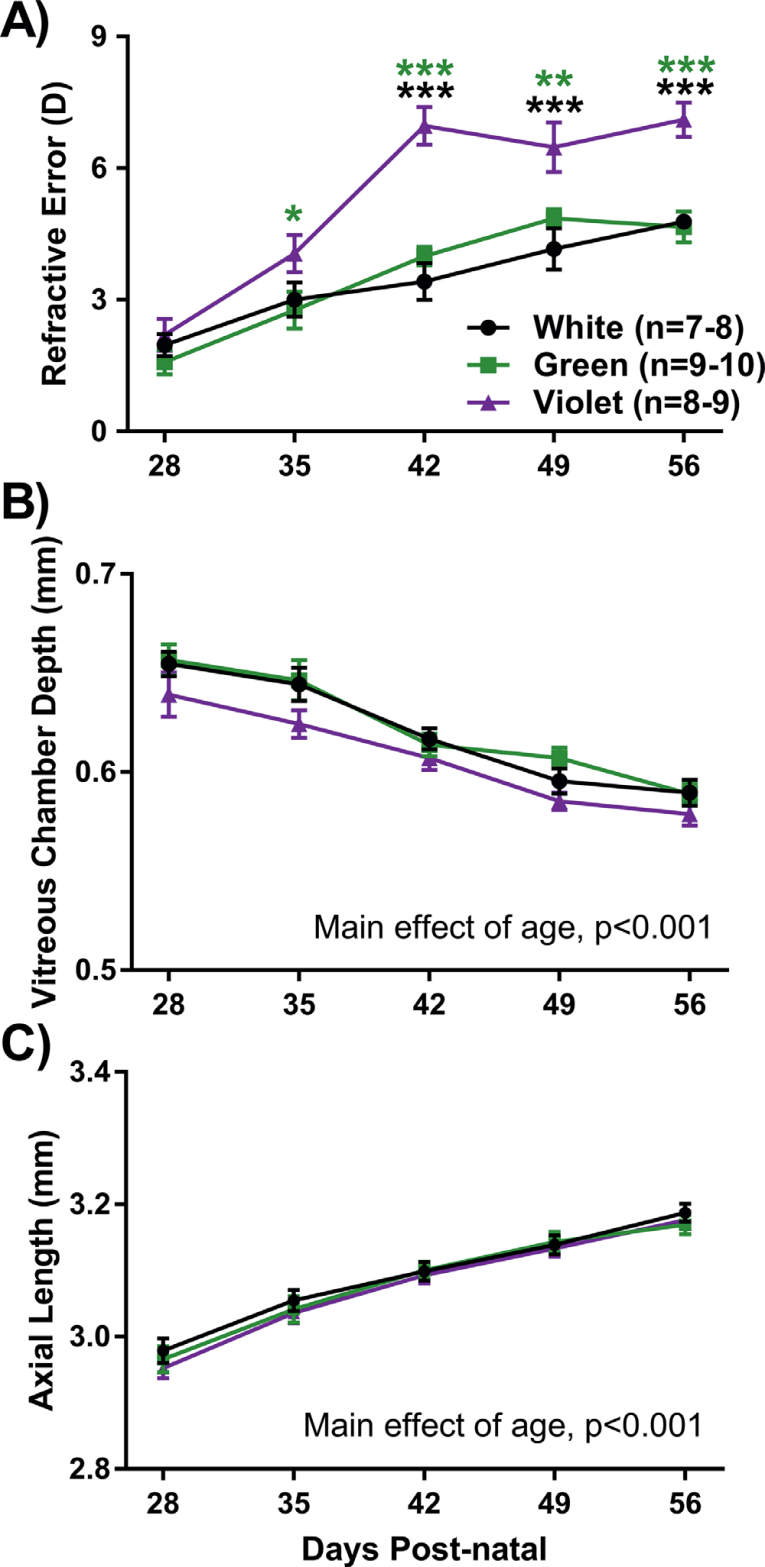
Refractive development of WT mice housed under three light conditions. (**A**) Violet light exposure significantly increased the degree of hyperopia after 1 week (repeated two-way ANOVA light by age interaction, F(8,132) = 4.14, *P* < 0.001). (**B**) There were no significant differences in vitreous chamber depth among the different light exposure groups (two-way repeated measures ANOVA main effect of light, F(2,132) = 0.6, *P* = 0.78). (**C**) There were no significant differences in axial length among the different light exposure groups (two-way repeated measures ANOVA main effect of light, F(2,132) = 0.2, *P* = 0.82). Post hoc comparisons are indicated by asterisks at each timepoint: *P < 0.05; **P < 0.01; ***P < 0.001. Asterisk color indicates comparisons with respective light group. Data displayed as mean ± SEM.

The refractive error of WT mice exposed to monochromatic violet light was significantly hyperopic compared with white light controls beginning at P42 and lasting until the end of the experiment at P56 (at P56 Violet: 7.00 ± 0.34 D, White: 4.67 ± 0.18 D, repeated two-way ANOVA interaction effect, F(8,132) = 4.14, *P* < 0.001, *post-hoc*: *P* < 0.001, Fig. 2A). In contrast, the refractions of mice housed in green light were not different from mice in white light across all timepoints (Fig. 2A). There were no significant differences in vitreous chamber depth or axial length between groups, as shown in Figure 2B and 2C, respectively, or other optical parameters such as corneal curvature and anterior chamber depth (see Supplementary Table S1).

### Violet Light Exposure Had Protective Effects on Lens-Induced Myopia

**Figure 3.**
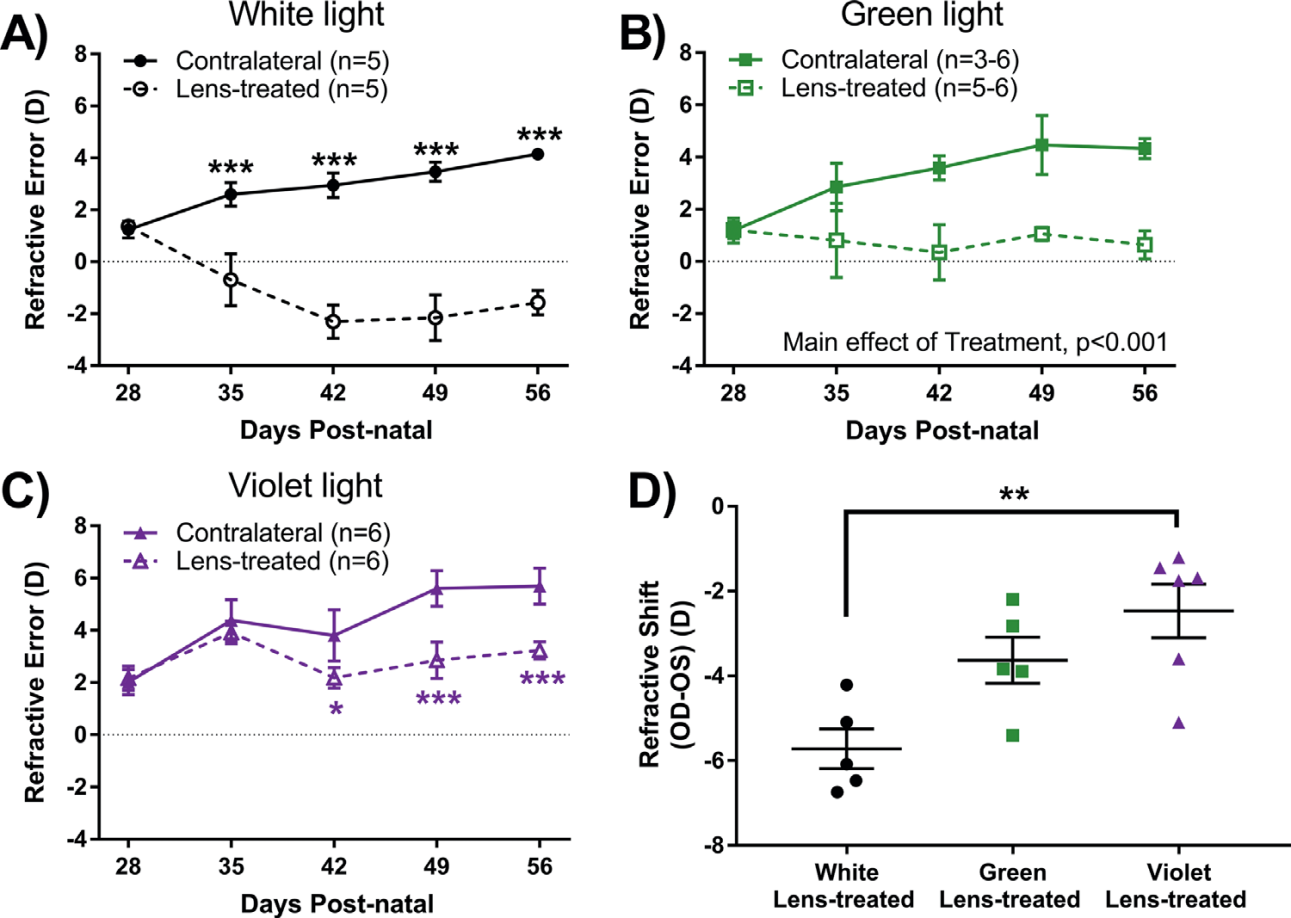
The eyes of WT mice in response to lens defocus under the three light conditions through four weeks of treatment. (**A**) Under white light, the refractive error of lens treated eyes was significantly myopic when compared to the contralateral eye beginning at P35 (two-way repeated measures ANOVA treatment by age interaction, F(4,49) = 9.21, *P* < 0.001). (**B**) Under green light, the refractive error of lens treated eyes was significantly myopic when compared to the contralateral eye (two-way repeated measures ANOVA main effect of treatment, F(1,46) = 218.94, *P* < 0.001). (**C**) Under violet light, the refractive error of lens treated eyes was significantly myopic when compared to the contralateral eye beginning at P42 (two-way repeated measures ANOVA treatment by age interaction, F(4,58) = 5.42, *P* < 0.001). (**D**) Mice exposed to violet light demonstrated a significant reduction in the average refractive shift at P56 (OD-OS) compared to the white-light group (one-way ANOVA, F(2,15) = 8.52, *P* = 0.004). Post hoc comparisons are indicated by asterisks at each timepoint: *P < 0.05; **P < 0.01; ***P < 0.001. Data displayed as mean ± SEM.

Although all WT eyes treated with lens defocus became significantly more myopic than the contralateral eye in all light conditions, the magnitude of the response was dependent on spectral composition. Under white light, lens-treated eyes became significantly more myopic than contralateral eyes across age (repeated two-way ANOVA interaction effect, F(4,49) = 9.21, *P* < 0.001, Fig. 3A), beginning at P35 (lens: −0.70 ± 1.0 D, contralateral: 2.59 ± 0.45 D, *P* < 0.001). Under green light, the lens-treated eye developed a more negative refractive error compared with the contralateral eye but did not reach a myopic refractive error (repeated two-way ANOVA main effect of treatment, F(1,46) = 218.94, *P* < 0.001, at P42 lens: 0.35 ± 1.06, contralateral: 3.58 ± 0.47, Fig. 3B). A similar effect was observed in lens-treated eyes under violet light with significant differences at P42 (lens: 2.19 ± 0.39 D, contralateral: 3.80 ± 0.98 D; repeated two-way ANOVA treatment by age interaction, F(4,58) = 5.42, *P* < 0.001, post hoc: *P* < 0.05, Fig. 3C). To directly compare the effect of light wavelength on LIM in WT animals, the refractive shift (lens-treated – contralateral) of the animals was compared at the final timepoint, P56. The refractive shift of mice exposed to violet light was significantly smaller when compared with mice exposed to white light (violet: −2.47 ± 0.63 D, white: −5.72 ± 0.47 D; two-way ANOVA, F(2,15) = 8.52, *P* = 0.004, Fig. 3D). There were no significant differences in the other ocular parameters of lens-treated WT mice across time regardless of light condition (see Supplementary Table S1).

### Dopamine Levels Were Unchanged by Spectral Exposure

**Figure 4.**
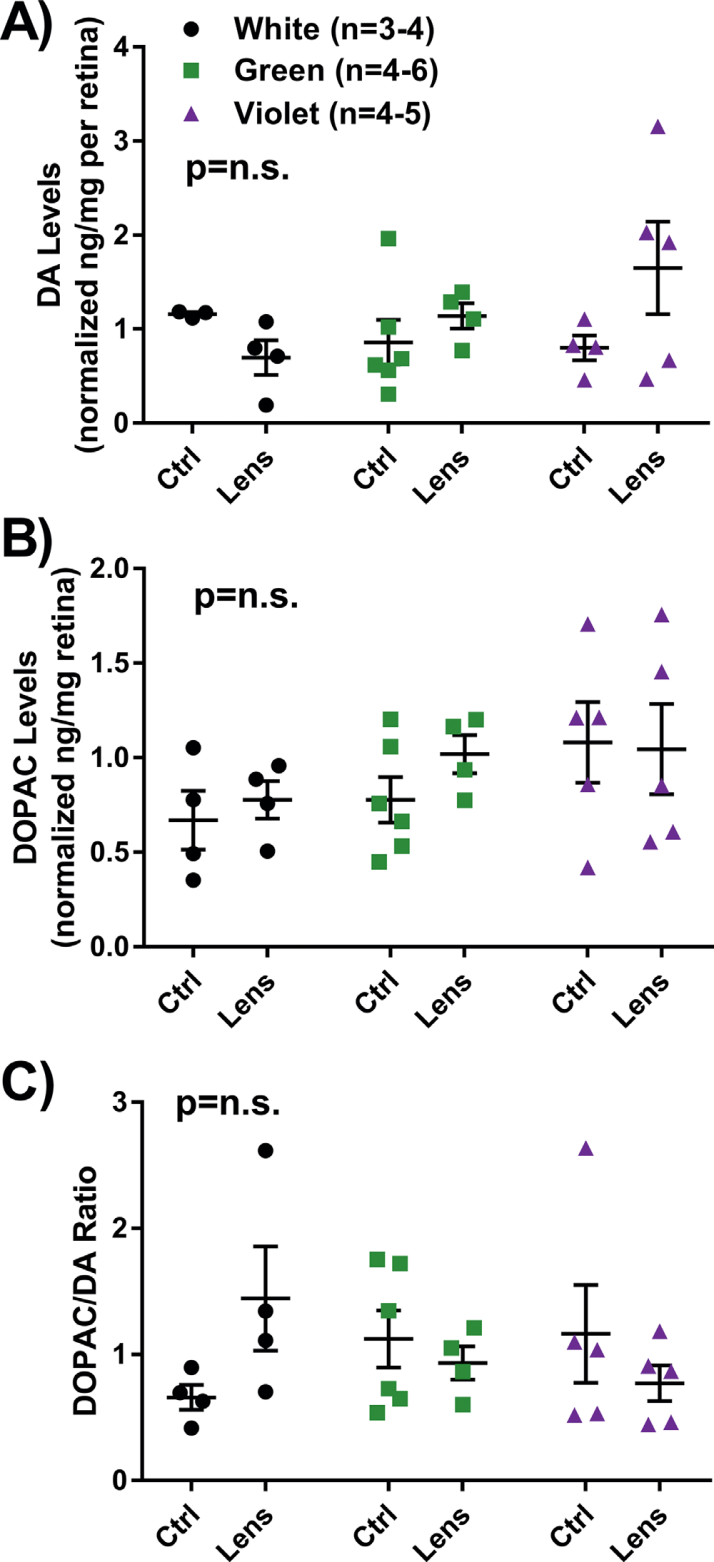
DA and DOPAC data obtained through HPLC from control and lens-treated WT retinas at P58. There were no significant differences between groups with respect to (**A**) dopamine levels, (**B**) DOPAC levels, or (**C**) the DOPAC/DA ratio, indicating DA turnover in the retina. Data displayed as mean ± SEM.

In WT control and lens-treated animals, there were no significant differences in dopamine levels within the retina resulting from light or lens treatment (Fig. 4A). Furthermore, DOPAC, one of the primary metabolites of DA, did not demonstrate any significant differences due to treatments (Fig. 4B). Last, the DOPAC/DA ratio, an indicator of DA turnover, was not significantly different between groups (Fig. 4C).

### Violet Light Exposure Does Not Induce Hyperopic Refractions in *Gnat2*^−/^^−^ Mice

**Figure 5.**
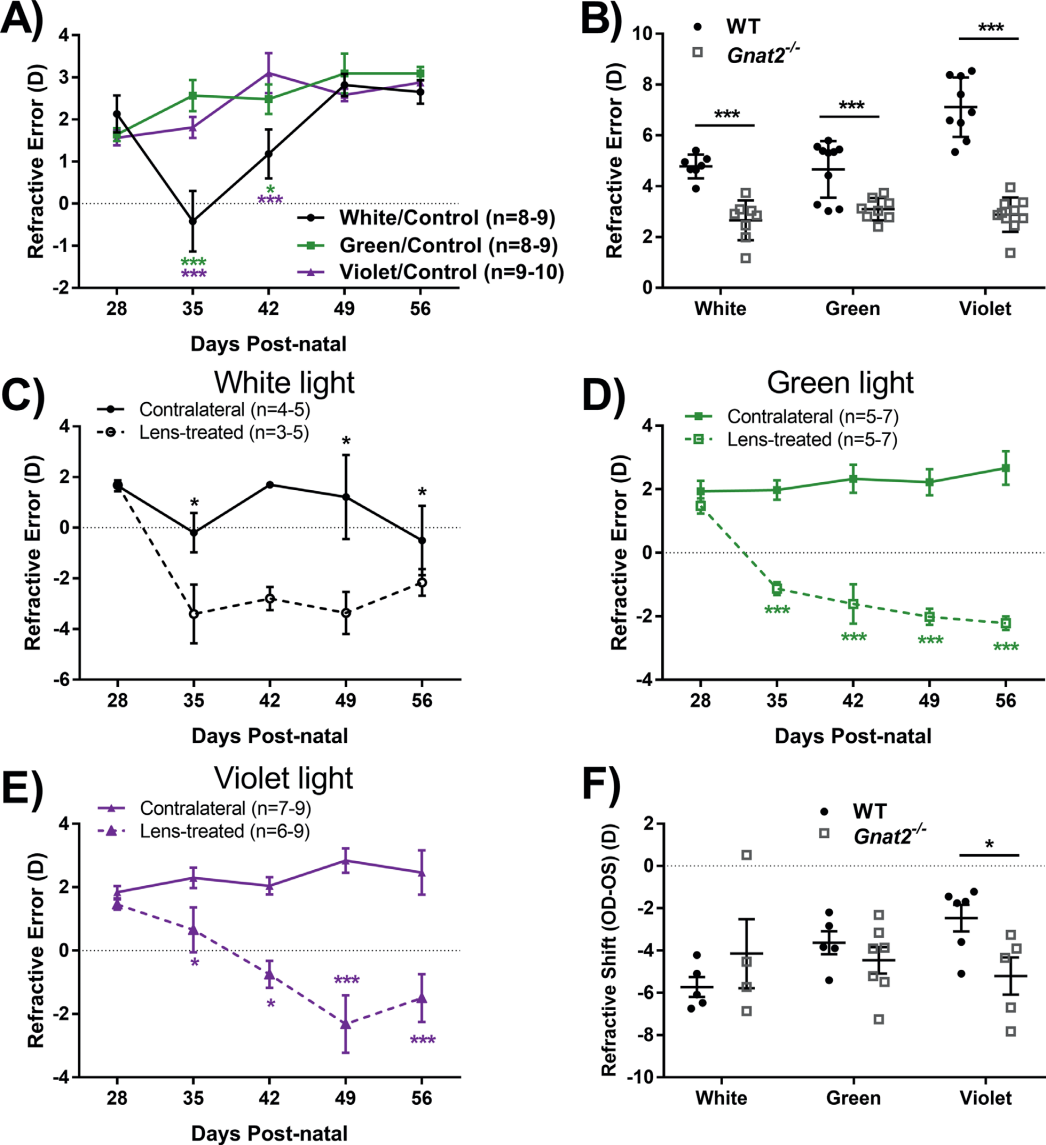
Loss of cone function in *Gnat2^−^^/^^−^* mice eliminated the effects of violet light. (**A**) *Gnat2^−^^/^^−^* mice exposed to white light were significantly more myopic than mice housed in monochromatic light at P35 and P42 (repeated two-way ANOVA interaction effect, F(8,131) = 5.83, *P* < 0.001). (**B**) Comparison of the refractive error of control WT and *Gnat2^−^^/^^−^* mice at P56; *Gnat2^−^^/-^* mice had significantly lower refractive errors across light groups compared to WT mice (two-way ANOVA main effect of strain, F(1,51) = 123.23, *P* < 0.001). (**C**) *Gnat2^−^^/^^−^* lens-treated eyes exposed to white light were significantly more myopic than contralateral eyes (repeated two-way ANOVA interaction effect, F(4,37) = 4.76, *P* = 0.021). (**D**) *Gnat2^−^^/^^−^* lens-treated eyes exposed to green light were significantly more myopic than contralateral eyes (two-way repeated measures ANOVA treatment by age interaction, F(4,49) = 22.15, *P* < 0.001). (**E**) *Gnat2^−^^/^^−^* lens-treated eyes exposed to violet light were significantly more myopic than contralateral eyes (repeated two-way ANOVA interaction effect, F(4,66) = 8.07, *P* < 0.001). (**F**) Comparison of the refractive shift of lens-treated WT and *Gnat2^−^^/^^−^* mice at P56; WT mice have a significantly reduced refractive shift compared to *Gnat2^−^^/^^−^* mice when exposed to violet light (two-way ANOVA strain by light interaction, F(2,31) = 3.44, *P* = 0.047). Post hoc comparisons are indicated by asterisks at each timepoint: *P < 0.05; ***P < 0.001. Asterisk color corresponds with respective light group. Data displayed as mean ± SEM.

*Gnat2^−^^/^^−^* animals with an absence of cone function did not develop increased levels of hyperopia when housed in violet light (violet: 1.81 ± 0.25 D). *Gnat2^−^^/^^−^* mice exposed to violet light had the same refractive errors with development as *Gnat2^−^^/^^−^* mice housed under green light (green: 2.57 ± 0.37 D). Unexpectedly, *Gnat2^−^^/^^−^* mice exposed to white light demonstrated significant myopic refractive changes after the first week of light treatment (at P35, white: −0.42 ± 0.72 D; repeated two-way ANOVA light by age interaction, F(8,131)=5.83, *P* < 0.001, *post-hoc*: *P* < 0.05, Fig. 5A). By P49, there were no significant differences in refractive errors between any light group. (See Supplementary Table S2 for additional ocular measurements.) The refractive error of *Gnat2^−^^/^^−^* mice was significantly lower than WT mice exposed to the same lighting conditions (two-way ANOVA main effect of strain, F(1,51) = 123.23, *P* < 0.001, Fig. 5B). Additionally, violet light did not induce increased levels of hyperopia in control *Gnat2^−^^/^^−^* mice as was demonstrated in the WT mice.

### Retinal Cone Pathways Required for the Protective Effects of Violet Light on Myopia

Loss of cone function also prevented the protective effects of violet light on LIM. *Gnat2^−^^/^^−^* lens-treated eyes developed the same magnitude of myopia under each light condition. Under white light, lens-treated eyes became significantly myopic after one week of lens defocus (contralateral: −0.20 ± 0.78 D, lens: −3.40 ± 1.16 D; repeated two-way ANOVA interaction effect, F(4,37) = 4.76, *P* = 0.021, post hoc: *P* < 0.05, Fig. 5C). Under green light, lens-treated eyes became significantly myopic after 1 week of defocus (contralateral: 1.97 ± 0.31 D, lens: −1.13 ± 0.20 D; repeated two-way ANOVA interaction effect, F(4,49) = 22.15, *P* < 0.001, post hoc: *P* < 0.001, Fig. 5D) and the difference persisted until the final timepoint. Similarly, when exposed to violet light, lens-treated eyes became significantly myopic beginning one week after defocus (contralateral: 2.30 ± 0.32 D, lens: 0.65 ± 0.70 D; repeated two-way ANOVA interaction effect, F(4,66) = 8.07, *P* < 0.001, post hoc: *P* < 0.05, Fig. 5E) and also lasted until the final timepoint.

At the final timepoint, P56, there were no differences in the amount of refractive shift among lens-treated *Gnat2^−^^/^^−^* mice regardless of light condition. Additionally, violet light exposure did not induce a protective effect in *Gnat2^−^^/^^−^* mice compared to WT mice (*Gnat2^−^^/^^−^*: −5.21 ± 0.88 D; WT: −2.47 ± 0.63 D; two-way ANOVA, F(2,31) = 3.44, *P* = 0.047, Fig. 5F).

### Evidence for Longer Axial Lengths in Lens-Treated Mice With Significant Myopic Shifts

**Figure 6.**
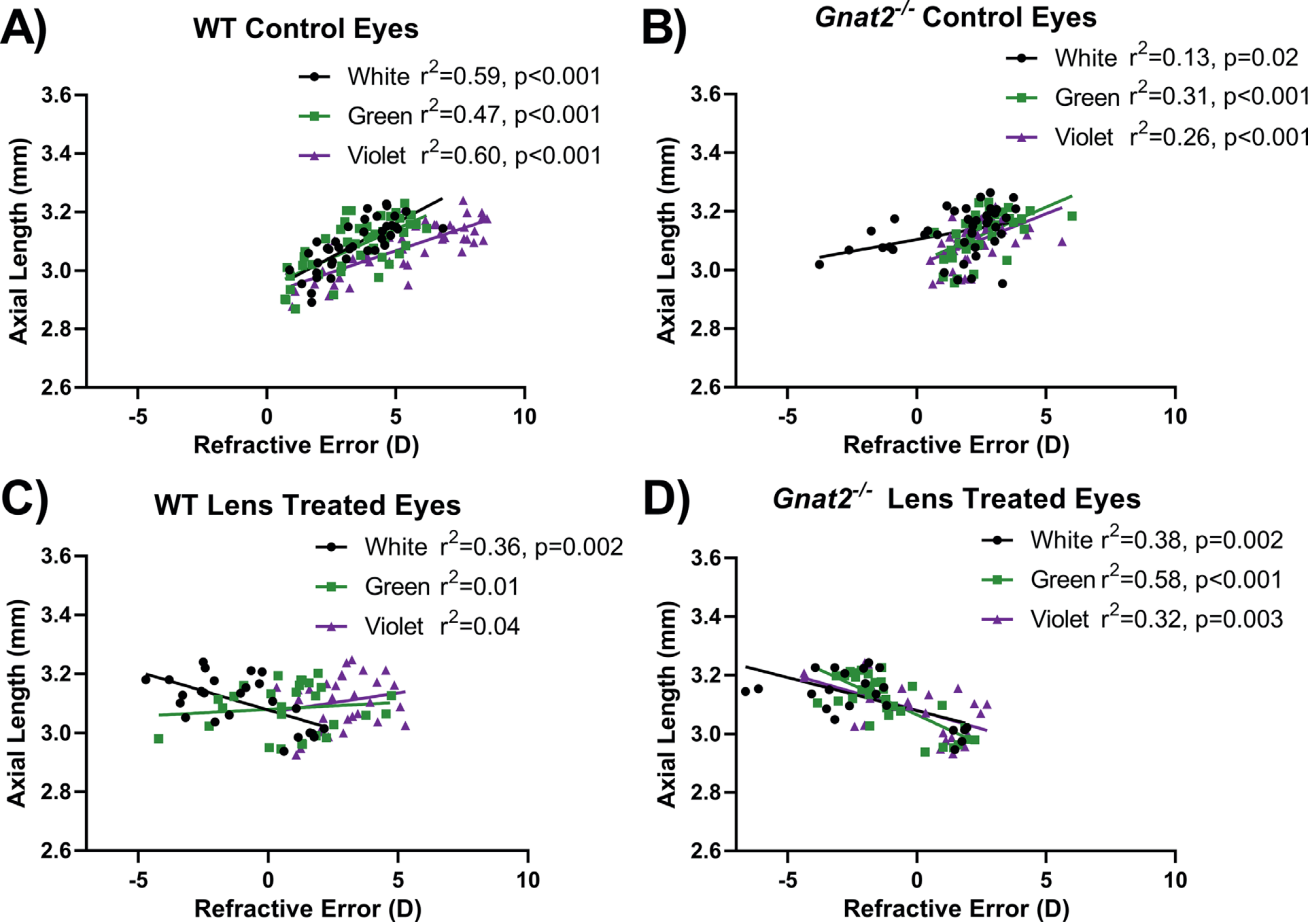
WT and *Gnat2^−^^/^^−^* mice with lens defocus show significant correlations with axial elongation and myopic refractions. (**A**) WT and (**B**) *Gnat2^−^^/^^−^* mice without lens defocus showed significant positive correlation for axial length versus refractive error across all ages tested. These trends can be explained by the normal developmental growth of the eye while the refractive error became more hyperopic with age. (**C**) WT LIM mice housed in white light showed a significant negative correlation such that longer axial lengths correlated with more myopic refractions. In contrast, WT LIM mice housed in green or violet light did not have significant correlations between axial length and refractive error. Thus, treatment groups with the largest myopic shift (white light) showed the greatest correlations between axial elongation and refractive error. (**D**) Likewise, in *Gnat2^−^^/^^−^* LIM mice, all light group showed significant negative correlations and large myopic shifts.

To further examine axial change in mice undergoing experimental myopia, we plotted refractive error versus axial length across all ages. In WT and *Gnat2^−^^/^^−^* mice without lens treatment housed in all three light conditions, more hyperopic refractive errors were significantly correlated with longer axial length (*r*^2^ = 0.47-0.60, *P* < 0.001 for WT mice and *r*^2^ = 0.13-0.31, *P* < 0.05 for *Gnat2^−^^/^^−^* mice, Fig. 6A and B). These positive correlations can be explained by normal murine eye development across age in which refractive errors became more hyperopic as the eye became larger (longer axial length; see Fig. 2). In contrast, lens defocus produced negative correlations in LIM mice that developed ≥4 D myopic shift. WT LIM mice housed in white light had more myopic refractions that significantly correlated with longer axial length (*r*^2^ = 0.36, *P* < 0.001, Fig. 6C). Likewise, *Gnat2^−^^/^^−^* LIM mice housed in all three lighting conditions showed more myopic refractions that correlated with longer axial length (*r*^2^ = 0.32-0.58, *P* < 0.002). WT LIM mice housed in green or violet light, with comparably smaller myopic shifts (see Fig. 5D), had linear regressions closer to zero.

## Discussion

Our study demonstrates the importance of spectral cues on murine refractive development, specifically the hyperopic effects of narrow-band, short-wavelength light exposure. Exposure to short-wavelength violet light also significantly reduced the myopic shift of eyes treated with lens defocus. These data suggest that short-wavelength light may protect against myopic growth in the mouse eye, potentially through a mechanism related to LCA, where short wavelengths focus in front of the retina to slow eye growth. Furthermore, *Gnat2^−^^/^^−^* mice with nonfunctional cones lacked the effects induced by short-wavelength light observed in WT mice, suggesting that cone pathways may be playing a role in the detection of monochromatic light and signaling of refractive eye growth. WT mice were partially protected from LIM when exposed to short-wavelength light, whereas *Gnat2^−^^/^^−^* mice exposed to short-wavelength light and LIM were not different than broad-spectrum white light-exposed animals.

The murine model of myopia has not been previously used to test the effects of monochromatic lighting on refractive development and myopia susceptibility. As a dichromat that is maximally sensitive to wavelengths of approximately 360 nm to 510 nm^39^^,^^52^, the spectral sensitivity of the mouse is unlike that of primates,^39^^,^^53^ but the retinal circuitry is similar. The properties of LCA in the mouse eye^36^ allow for different monochromatic lights to influence refractive growth in a wavelength dependent manner. Within 2 weeks, short-wavelength light significantly altered the refractive development of WT mice. WT mice became more hyperopic in response to short-wavelength light exposure. The UV- and M-cones of the mouse would be responsive to the short-wavelength light used in this study (see Fig. 1).^33^^,^^34^^,^^39^ The refractive development of mice exposed to medium-wavelength and broad-spectrum white light are not significantly different. This may be explained by the fact that both light sources are likely to activate M-cone and rod pathways.^38^^,^^52^

The main optical parameters that changed in direct relation to the refractive error of the mice was axial length. In the present study, axial length was found to negatively correlate with refractive error in mice that developed significant myopic shifts such that more myopic refractive errors were associated with longer axial lengths (Fig. 6). Axial elongation is a hallmark of myopia^2^ that is often undetectable in the mouse model of myopia when making direct comparisons of axial length between myopic and nonmyopic eyes.^48^ This is likely due to the sensitivity needed to measure ∼5 µm difference in axial length for each 1 D of refractive shift.^47^^,^^48^ While all LIM eyes showed a change in linear regression slope from the control eyes, only groups with larger myopic shifts showed a significant negative slope (Fig. 6). Other ocular parameters were measured, but they were not significantly different between light conditions and do not further explain the differences in refraction.

Furthermore, short-wavelength light was found to be protective against LIM in WT mice. Lens-treated eyes became significantly more myopic than the contralateral eyes, but the magnitude of the refractive shift was different across spectral conditions. Here, animals in short-wavelength light demonstrated a significantly reduced refractive shift compared to animals in broad-spectrum white light. Previous studies in guinea pigs and chicks have also provided evidence that short-wavelength light reduced the severity of experimental myopia.^27^^,^^28^^,^^31^ Although the wavelengths of light in these studies vary, it is clear that relatively shorter wavelengths can protect against LIM. This protection could likely be due to the interaction of the lens treatment and the short wavelength light that refracts to a greater degree upon transmission, altering the focal plane to a more anterior position and potentially slowing refractive eye growth. It is also noteworthy that the broad-spectrum white light used here did not contain wavelengths less than ∼415 nm and did not promote antimyopigenic signals in the eye. More research is needed to determine if a broader spectrum stimulus that included UV wavelengths would provide some protection compared with monochromatic light.

In this study, there were no differences in the dopamine or DOPAC content of the retina. In contrast, chicks exposed to monochromatic short wavelength light with lens defocus showed less myopia and a significant increase in DA release.^31^ However, the significant changes in DA were found in a much shorter exposure time to the light (30 minutes-5 days). After 4 weeks of light exposure used in this study, it is possible that DA and DOPAC levels returned to a normalized level because of homeostasis. Future studies could evaluate the effect of monochromatic light exposure on dopamine activity immediately after light exposure or increase the sample size to detect persistent changes in DA levels.

Prior to this study, it was unclear which photoreceptor type may contribute to the protective effects of violet light. By using *Gnat2^−^^/^^−^* animals we showed that mice exposed to short-wavelength light lacked the hyperopic increase under normal visual conditions and did not show a reduced response to lens defocus. These findings indicate that UV-cones in the circuitry of the mouse retina could underlie the protection from short-wavelength light. Interestingly, *Gnat2^−^^/^^−^* mice under short-wavelength light did respond to the lens defocus, indicating that the retina could detect blur. One study has shown that the rods of *Gnat2^−^^/^^−^* mice become sensitive to UV-A light, which could be one possible explanation for the detection of defocus.^38^ In addition, although more sensitive to blue light, it is possible that melanopsin (Opn4)^54^ or neuropsin (Opn5)^55^^,^^56^ could also detect the violet light used. Furthermore, there is evidence that melanopsin is involved in the refractive development of the mouse eye.^57^ Additional studies could explore the contributions of these other photoreceptor types as well as UV-cones specifically for their contributions to the effects of violet light on refractive eye growth.

*Gnat2^−^^/^^−^* mice have recently been reported to have similar refractive development across age and an increased response to form deprivation compared to age-matched WT mice.^58^ This study found *Gnat2^−^^/^^−^* mice to have significantly less hyperopia than WT controls (Fig. 5B). These differences may be due to the chromatic spectrum of fluorescent lighting (spectral peaks at 430, 545, and 610 nm with 70% of the spectral power greater than 530 nm^59^) compared with the white LED light source (420-680 nm) used in this study. Furthermore, *Gnat2^−^^/^^−^* mice were found to have increased susceptibility to form deprivation compared with WT mice.^58^ In this study, *Gnat2^−^^/^^−^* mice had a similar response to LIM in all three lighting conditions, departing from the response from WT mice that showed reduced LIM under violet light (Fig. 5F). Further research is needed to determine if these differences reveal that cone pathways are modulating refractive development differentially, depending on illumination levels and the type of myopigenic stimuli.

The data presented demonstrate important findings related to refractive development and monochromatic light exposure but must be interpreted with limitations in mind. Refractions from the small mouse eye are likely not the absolute refractive error of the eye due to the small eye artifact.^47^^,^^60^ However, since the same method was used to measure refractive error across all groups, the values can be relatively compared. In addition, the peak spectral output of the short-wavelength light source used here is not equal to the peak sensitivity of the UV-cone. Similarly, the relative intensity of the monochromatic light sources used were calibrated in relation to the intensity of the broad-spectrum white light control. The monochromatic light conditions would likely activate the expected cone photoreceptors but not equally due to differences in the cell number of M- and UV-cones and the increased spectral sensitivity of UV- versus M-cones (Fig. 1). Additionally, other photosensitive cells in the retina with overlapping spectral sensitivities cannot be ruled out and should be investigated further with additional mutant mouse models. Therefore, a proposed retinal pathway of protection from LIM using short-wavelength light should be interpreted with caution until photoreceptor activation is better controlled.

In conclusion, short-wavelength light induced hyperopia in wild-type mouse eyes with normal vision. Additionally, short-wavelength light attenuated myopic shifts in wild-type mice with lens defocus relative to broad-spectrum white light. Using a mouse model with cone dysfunction, we found that the effects of short-wavelength light were diminished in both normal-vision animals as well as animals treated with lens defocus. Future studies are needed to understand the role of short-wavelength sensitive cones in mouse myopia research, as well as the role of other retinal signaling mechanisms in the interpretation of chromatic blur.

## Supplementary Material

Supplement 1

## References

[1] Foster PJ, Jiang Y Epidemiology of myopia. *Eye*. 2014; 28: 202–208. 24406412 10.1038/eye.2013.280 PMC3930282

[2] Norton TT, Siegwart JT Jr Animal models of emmetropization: matching axial length to the focal plane. *J Am Optom Assoc*. 1995; 66: 405–414. 7560727

[3] Jin JX, Hua WJ, Jiang X, et al. Effect of outdoor activity on myopia onset and progression in school-aged children in northeast China: the Sujiatun Eye Care Study. *BMC Ophthalmol*. 2015; 15: 73. 26152123 10.1186/s12886-015-0052-9 PMC4495846

[4] Read SA, Collins MJ, Vincent SJ Light exposure and physical activity in myopic and emmetropic children. *Optom Vis Sci*. 2014; 91: 330–341. 24413273 10.1097/OPX.0000000000000160

[5] Rose KA, Morgan IG, Ip J, et al. Outdoor activity reduces the prevalence of myopia in children. *Ophthalmology*. 2008; 115: 1279–1285. 18294691 10.1016/j.ophtha.2007.12.019

[6] Norton TT, Siegwart JT Jr Light levels, refractive development, and myopia–a speculative review. *Exp Eye Res*. 2013; 114: 48–57. 23680160 10.1016/j.exer.2013.05.004 PMC3742693

[7] Thorne HC, Jones KH, Peters SP, Archer SN, Dijk DJ Daily and seasonal variation in the spectral composition of light exposure in humans. *Chronobio Int*. 2009; 26: 854–866. 10.1080/07420520903044315 19637047

[8] Krutmann J, Behar-Cohen F, Baillet G, et al. Towards standardization of UV eye protection: what can be learned from photodermatology? *Photodermatol Photoimmunol Photomed*. 2014; 30: 128–136. 24303877 10.1111/phpp.12089

[9] Charman WN, Jennings JA Objective measurements of the longitudinal chromatic aberration of the human eye. *Vision Re**s*. 1976; 16: 999–1005. 948891 10.1016/0042-6989(76)90232-7

[10] Collins MJ, Wildsoet CF, Atchison DA Monochromatic aberrations and myopia. *Vision Res*. 1995; 35: 1157–1163. 7610577 10.1016/0042-6989(94)00236-f

[11] Mandelman T, Sivak JG Longitudinal chromatic aberration of the vertebrate eye. *Vision Res*. 1983; 23: 1555–1559. 6666057 10.1016/0042-6989(83)90169-4

[12] Seidemann A, Schaeffel F Effects of longitudinal chromatic aberration on accommodation and emmetropization. *Vision Res*. 2002; 42: 2409–2417. 12367740 10.1016/s0042-6989(02)00262-6

[13] Wildsoet CF, Howland HC, Falconer S, Dick K Chromatic aberration and accommodation: their role in emmetropization in the chick. *Vision Res*. 1993; 33: 1593–1603. 8236848 10.1016/0042-6989(93)90026-s

[14] Charman WN Aberrations and myopia. *Ophthal Physiol Optics*. 2005; 25: 285–301. 10.1111/j.1475-1313.2005.00297.x 15953113

[15] Rucker FJ, Kruger PB Cone contributions to signals for accommodation and the relationship to refractive error. *Vision Res*. 2006; 46: 3079–3089. 16782165 10.1016/j.visres.2006.04.009

[16] Hung LF, Arumugam B, She Z, Ostrin L, Smith EL 3rd Narrow-band, long-wavelength lighting promotes hyperopia and retards vision-induced myopia in infant rhesus monkeys. *Exp Eye Res*. 2018; 176: 147–160. 29981345 10.1016/j.exer.2018.07.004 PMC6215717

[17] Smith EL 3rd, Hung LF, Arumugam B, Holden BA, Neitz M, Neitz J Effects of long-wavelength lighting on refractive development in infant rhesus monkeys. *Invest Ophthalmol Visual Sci*. 2015; 56: 6490–6500. 26447984 10.1167/iovs.15-17025 PMC4604957

[18] Gawne TJ, Ward AH, Norton TT Long-wavelength (red) light produces hyperopia in juvenile and adolescent tree shrews. *Vision Res*. 2017; 140: 55–65. 28801261 10.1016/j.visres.2017.07.011 PMC5723538

[19] Rucker FJ, Wallman J Cone signals for spectacle-lens compensation: differential responses to short and long wavelengths. *Vision Res*. 2008; 48: 1980–1991. 18585403 10.1016/j.visres.2008.06.003 PMC2790044

[20] Rucker FJ, Wallman J Chick eyes compensate for chromatic simulations of hyperopic and myopic defocus: evidence that the eye uses longitudinal chromatic aberration to guide eye-growth. *Vision Res*. 2009; 49: 1775–1783. 19383509 10.1016/j.visres.2009.04.014 PMC2779109

[21] Kroger RH, Fernald RD Regulation of eye growth in the African cichlid fish Haplochromis burtoni. *Vision Res*. 1994; 34: 1807–1814. 7941383 10.1016/0042-6989(94)90305-0

[22] Kroger RH, Wagner HJ The eye of the blue acara (Aequidens pulcher, Cichlidae) grows to compensate for defocus due to chromatic aberration. *J Comp Physiol A*. 1996; 179: 837–842. 8956500 10.1007/BF00207362

[23] Liu R, Qian YF, He JC, et al. Effects of different monochromatic lights on refractive development and eye growth in guinea pigs. *Exp Eye Res*. 2011; 92: 447–453. 21396363 10.1016/j.exer.2011.03.003

[24] Long Q, Chen D, Chu R Illumination with monochromatic long-wavelength light promotes myopic shift and ocular elongation in newborn pigmented guinea pigs. *Cutaneous Ocular Toxicol*. 2009; 28: 176–180. 10.3109/15569520903178364 19888887

[25] Qian YF, Liu R, Dai JH, Chen MJ, Zhou XT, Chu RY Transfer from blue light or green light to white light partially reverses changes in ocular refraction and anatomy of developing guinea pigs. *J Vision*. 2013; 13. 10.1167/13.11.16 24071588

[26] Zou L, Zhu X, Liu R, et al. Effect of altered retinal cones/opsins on refractive development under monochromatic lights in guinea pigs. *J Ophthalmol*. 2018; 2018: 9197631. 29675275 10.1155/2018/9197631 PMC5838468

[27] Torii H, Ohnuma K, Kurihara T, Tsubota K, Negishi K Violet light transmission is related to myopia progression in adult high myopia. *Scient Rep*. 2017; 7: 14523. 10.1038/s41598-017-09388-7 PMC5674003 29109514

[28] Jiang L, Zhang S, Schaeffel F, et al. Interactions of chromatic and lens-induced defocus during visual control of eye growth in guinea pigs (Cavia porcellus). *Vision Res*. 2014; 94: 24–32. 24216006 10.1016/j.visres.2013.10.020

[29] Feldkaemper M, Schaeffel F An updated view on the role of dopamine in myopia. *Exp Eye Res*. 2013; 114: 106–119. 23434455 10.1016/j.exer.2013.02.007

[30] Z hou X, Pardue MT, Iuvone PM, Qu J Dopamine signaling and myopia development: what are the key challenges. *Prog Retin Eye Res*. 2017; 61: 60–71. 28602573 10.1016/j.preteyeres.2017.06.003 PMC5653403

[31] Wang M, Schaeffel F, Jiang B, Feldkaemper M Effects of light of different spectral composition on refractive development and retinal dopamine in chicks. *Invest Ophthalmol Visual Sci*. 2018; 59: 4413–4424. 30193312 10.1167/iovs.18-23880

[32] Jacobs GH, Deegan JF 2nd Spectral sensitivity, photopigments, and color vision in the guinea pig (Cavia porcellus). *Behav Neurosci*. 1994; 108: 993–1004. 7826522 10.1037//0735-7044.108.5.993

[33] Jacobs GH, Neitz J, Deegan JF 2nd Retinal receptors in rodents maximally sensitive to ultraviolet light. *Nature*. 1991; 353: 655–656. 1922382 10.1038/353655a0

[34] Nikonov SS, Kholodenko R, Lem J, Pugh EN Jr Physiological features of the S- and M-cone photoreceptors of wild-type mice from single-cell recordings. *J Gen Physiol*. 2006; 127: 359–374. 16567464 10.1085/jgp.200609490 PMC2151510

[35] Xu X, Quiambao AB, Roveri L, et al. Degeneration of cone photoreceptors induced by expression of the Mas1 protooncogene. *Exp Neurol*. 2000; 163: 207–219. 10785460 10.1006/exnr.2000.7370

[36] Geng Y, Schery LA, Sharma R, et al. Optical properties of the mouse eye. *Biomed Optics Exp*. 2011; 2: 717–738. 10.1364/BOE.2.000717 PMC3072116 21483598

[37] Chang B, Dacey MS, Hawes NL, et al. Cone photoreceptor function loss-3, a novel mouse model of achromatopsia due to a mutation in Gnat2. *Invest Ophthalmol Vis Sci*. 2006; 47: 5017–5021. 17065522 10.1167/iovs.05-1468

[38] Wang YV, Weick M, Demb JB Spectral and temporal sensitivity of cone-mediated responses in mouse retinal ganglion cells. *J Neurosc*. 2011; 31: 7670–7681. 10.1523/JNEUROSCI.0629-11.2011 PMC3122925 21613480

[39] Jacobs GH, Williams GA, Fenwick JA Influence of cone pigment coexpression on spectral sensitivity and color vision in the mouse. *Vision Res*. 2004; 44: 1615–1622. 15135998 10.1016/j.visres.2004.01.016

[40] Jacobs GH, Williams GA Contributions of the mouse UV photopigment to the ERG and to vision. *Doc Ophthalmol*. 2007; 115: 137–144. 17479214 10.1007/s10633-007-9055-z

[41] Umino Y, Solessio E, Barlow RB Speed, spatial, and temporal tuning of rod and cone vision in mouse. *J Neurosci*. 2008; 28: 189–198. 18171936 10.1523/JNEUROSCI.3551-07.2008 PMC2847259

[42] Pardue MT, Faulkner AE, Fernandes A, et al. High susceptibility to experimental myopia in a mouse model with a retinal on pathway defect. *Invest Ophthalmol Vis Sci*. 2008; 49: 706–712. 18235018 10.1167/iovs.07-0643 PMC2752325

[43] Park H, Qazi Y, Tan C, et al. Assessment of axial length measurements in mouse eyes. *Optom Vis Sci*. 2012; 89: 296–303. 22246334 10.1097/OPX.0b013e31824529e5 PMC3310398

[44] Park H, Tan CC, Faulkner A, et al. Retinal degeneration increases susceptibility to myopia in mice. *Mol Vision*. 2013; 19: 2068–2079. PMC3786452 24146540

[45] Schaeffel F, Burkhardt E, Howland HC, Williams RW Measurement of refractive state and deprivation myopia in two strains of mice. *Optom Vis Sci*. 2004; 81: 99–110. 15127929 10.1097/00006324-200402000-00008

[46] Schaeffel F Test systems for measuring ocular parameters and visual function in mice. *Front Bioscience*. 2008; 13: 4904–4911. 10.2741/3049 18508555

[47] Schmucker C, Schaeffel F A paraxial schematic eye model for the growing C57BL/6 mouse. *Vision Res*. 2004; 44: 1857–1867. 15145680 10.1016/j.visres.2004.03.011

[48] Pardue MT, Stone RA, Iuvone PM Investigating mechanisms of myopia in mice. *Exp Eye Res*. 2013; 114: 96–105. 23305908 10.1016/j.exer.2012.12.014 PMC3898884

[49] Faulkner AE, Kim MK, Iuvone PM, Pardue MT Head-mounted goggles for murine form deprivation myopia. *J Neurosci Methods*. 2007; 161: 96–100. 17126909 10.1016/j.jneumeth.2006.10.011

[50] Witkovsky P Dopamine and retinal function. *Doc Ophthalmol*. 2004; 108: 17–40. 15104164 10.1023/b:doop.0000019487.88486.0a

[51] Song CH, Fan X, Exeter CJ, Hess EJ, Jinnah HA Functional analysis of dopaminergic systems in a DYT1 knock-in mouse model of dystonia. *Neurobiol Dis*. 2012; 48: 66–78. 22659308 10.1016/j.nbd.2012.05.009 PMC3498628

[52] Rocha FA, Gomes BD, Silveira LC, et al. Spectral sensitivity measured with electroretinogram using a constant response method. *PloS One*. 2016; 11: e0147318. 26800521 10.1371/journal.pone.0147318 PMC4723306

[53] Schnapf JL, Kraft TW, Nunn BJ, Baylor DA Spectral sensitivity of primate photoreceptors. *Visual Neurosci*. 1988; 1: 255–261. 10.1017/s0952523800001917 3154798

[54] Hankins MW, Peirson SN, Foster RG Melanopsin: an exciting photopigment. *Trends Neurosci*. 2008; 31: 27–36. 18054803 10.1016/j.tins.2007.11.002

[55] Buhr ED, Yue WW, Ren X, et al. Neuropsin (OPN5)-mediated photoentrainment of local circadian oscillators in mammalian retina and cornea. *Proc Nat**l Acad Sci U**S**A*. 2015; 112: 13093–13098. 26392540 10.1073/pnas.1516259112 PMC4620855

[56] Kojima D, Mori S, Torii M, Wada A, Morishita R, Fukada Y UV-sensitive photoreceptor protein OPN5 in humans and mice. *PloS One*. 2011; 6: e26388. 22043319 10.1371/journal.pone.0026388 PMC3197025

[57] Chakraborty R, Lee DC, Landis EG, et al. Melanopsin knock-out mice have abnormal refractive development and increased susceptibility to form-deprivation myopia. *Invest Ophthalmol Vis Sci*. 2015; 56: 5843–5843.

[58] Chakraborty R, Yang V, Park HN, et al. Lack of cone mediated retinal function increases susceptibility to form-deprivation myopia in mice. *Exp Eye Res*. 2019; 180: 226–230. 30605665 10.1016/j.exer.2018.12.021 PMC6642639

[59] Bergen MA, Park HN, Chakraborty R, et al. Altered refractive development in mice with reduced levels of retinal dopamine. *Invest Ophthalmol Vis Sci*. 2016; 57: 4412–4419. 27750284 10.1167/iovs.15-17784 PMC5015967

[60] Glickstein M, Millodot M Retinoscopy and eye size. *Science (New York, NY)*. 1970; 168: 605–606. 10.1126/science.168.3931.605 5436596

