# Supplementary material for: Short-Wavelength (Violet) Light Protects Mice From Myopia Through Cone Signaling
Source: Invest Ophthalmol Vis Sci. 2020 Feb 12;61(2):13. doi: 10.1167/iovs.61.2.13 (PMC7326482; doi:10.1167/iovs.61.2.13)
Supplement: Supplement 1 [file iovs-61-2-13_s001.pdf]

Supplementary Material
Strickland et al., Violet light protects mice from lens induced myopia through cone signaling,

Table S1. WT ocular parameters represented as mean ± SEM.

WHITE

Ocular Parameter	Days Post-natal	28		35		42		49		56	
Radius of Corneal Curvature (mm)	Control (n=6-8)	1.42	± 0.03	1.47	± 0.03	1.51	± 0.03	1.52	± 0.02	1.54	± 0.02
	Lens-treated (n=4-5)	1.48	± 0.01	1.49	± 0.00	1.54	± 0.01	1.55	± 0.01	1.58	± 0.01
Corneal Thickness (mm)	Control (n=8)	0.08	± 0.00	0.08	± 0.00	0.08	± 0.00	0.08	± 0.00	0.08	± 0.00
	Lens-treated (n=5)	0.08	± 0.00	0.08	± 0.00	0.08	± 0.00	0.08	± 0.00	0.08	± 0.00
Anterior Chamber Depth (mm)	Control (n=8)	0.32	± 0.01	0.34	± 0.00	0.35	± 0.00	0.37	± 0.00	0.38	± 0.00
	Lens-treated (n=5)	0.32	± 0.00	0.34	± 0.01	0.36	± 0.01	0.38	± 0.01	0.39	± 0.00
Lens Thickness (mm)	Control (n=8)	1.75	± 0.01	1.81	± 0.01	1.87	± 0.01	1.92	± 0.01	1.96	± 0.01
	Lens-treated (n=5)	1.74	± 0.01	1.82	± 0.01	1.88	± 0.01	1.92	± 0.00	1.96	± 0.01
Vitreous Chamber Depth (mm)	Control (n=8)	0.65	± 0.01	0.64	± 0.01	0.62	± 0.01	0.60	± 0.01	0.59	± 0.01
	Lens-treated (n=5)	0.66	± 0.01	0.63	± 0.01	0.62	± 0.01	0.61	± 0.01	0.60	± 0.00
Retinal Thickness (mm)	Control (n=8)	0.18	± 0.00	0.18	± 0.00	0.17	± 0.00	0.18	± 0.00	0.18	± 0.00
	Lens-treated (n=5)	0.18	± 0.00	0.18	± 0.00	0.18	± 0.00	0.18	± 0.00	0.18	± 0.00
Axial Length (mm)	Control (n=8)	2.98	± 0.02	3.05	± 0.02	3.10	± 0.01	3.14	± 0.01	3.19	± 0.01
	Lens-treated (n=5)	2.98	± 0.01	3.05	± 0.01	3.12	± 0.01	3.16	± 0.01	3.21	± 0.01

GREEN

Ocular Parameter	Days Post-natal	28		35		42		49		56	
Radius of Corneal Curvature (mm)	Control (n=10)	1.41	± 0.03	1.47	± 0.02	1.50	± 0.03	1.52	± 0.02	1.53	± 0.02
	Lens-treated (n=6)	1.45	± 0.02	1.50	± 0.01	1.53	± 0.01	1.54	± 0.01	1.57	± 0.01
Corneal Thickness (mm)	Control (n=9-10)	0.08	± 0.00	0.07	± 0.00	0.08	± 0.00	0.08	± 0.00	0.08	± 0.00
	Lens-treated (n=5-6)	0.08	± 0.00	0.08	± 0.00	0.08	± 0.00	0.07	± 0.00	0.08	± 0.00
Anterior Chamber Depth (mm)	Control (n=9-10)	0.31	± 0.01	0.34	± 0.01	0.36	± 0.00	0.37	± 0.00	0.37	± 0.00
	Lens-treated (n=5-6)	0.31	± 0.00	0.34	± 0.00	0.36	± 0.00	0.38	± 0.00	0.39	± 0.01
Lens Thickness (mm)	Control (n=9-10)	1.74	± 0.01	1.81	± 0.01	1.87	± 0.01	1.91	± 0.01	1.95	± 0.01
	Lens-treated (n=5-6)	1.74	± 0.01	1.80	± 0.01	1.87	± 0.01	1.91	± 0.01	1.94	± 0.01
Vitreous Chamber Depth (mm)	Control (n=9-10)	0.66	± 0.01	0.65	± 0.01	0.61	± 0.01	0.61	± 0.01	0.59	± 0.01
	Lens-treated (n=5-6)	0.64	± 0.01	0.63	± 0.00	0.62	± 0.00	0.61	± 0.01	0.58	± 0.01
Retinal Thickness (mm)	Control (n=9-10)	0.18	± 0.00	0.17	± 0.00	0.18	± 0.00	0.18	± 0.00	0.17	± 0.00
	Lens-treated (n=5-6)	0.19	± 0.00	0.18	± 0.00	0.18	± 0.00	0.18	± 0.00	0.18	± 0.00
Axial Length (mm)	Control (n=9-10)	2.97	± 0.02	3.04	± 0.02	3.10	± 0.01	3.14	± 0.02	3.17	± 0.01
	Lens-treated (n=5-6)	2.97	± 0.01	3.04	± 0.01	3.11	± 0.01	3.15	± 0.01	3.18	± 0.01

VIOLET

Ocular Parameter	Days Post-natal	28		35		42		49		56	
Radius of Corneal Curvature (mm)	Control (n=8-9)	1.43	± 0.02	1.48	± 0.02	1.51	± 0.02	1.53	± 0.02	1.54	± 0.01
	Lens-treated (n=5-6)	1.49	± 0.02	1.50	± 0.01	1.53	± 0.01	1.54	± 0.01	1.57	± 0.02
Corneal Thickness (mm)	Control (n=9)	0.08	± 0.00	0.08	± 0.00	0.08	± 0.00	0.08	± 0.00	0.08	± 0.00
	Lens-treated (n=6)	0.08	± 0.00	0.08	± 0.00	0.07	± 0.00	0.08	± 0.00	0.08	± 0.00
Anterior Chamber Depth (mm)	Control (n=9)	0.31	± 0.00	0.34	± 0.00	0.35	± 0.00	0.36	± 0.00	0.38	± 0.00
	Lens-treated (n=6)	0.32	± 0.01	0.35	± 0.00	0.37	± 0.00	0.38	± 0.00	0.39	± 0.00
Lens Thickness (mm)	Control (n=9)	1.74	± 0.01	1.80	± 0.01	1.87	± 0.01	1.92	± 0.01	1.96	± 0.01
	Lens-treated (n=6)	1.75	± 0.01	1.82	± 0.01	1.89	± 0.01	1.92	± 0.01	1.96	± 0.01
Anterior Chamber Depth (mm)	Control (n=9)	0.63	± 0.01	0.62	± 0.01	0.61	± 0.01	0.59	± 0.00	0.58	± 0.01
	Lens-treated (n=6)	0.66	± 0.01	0.62	± 0.01	0.61	± 0.01	0.60	± 0.01	0.58	± 0.00
Retinal Thickness (mm)	Control (n=9)	0.18	± 0.00	0.18	± 0.00	0.18	± 0.00	0.18	± 0.00	0.18	± 0.00
	Lens-treated (n=6)	0.18	± 0.00	0.18	± 0.00	0.18	± 0.00	0.18	± 0.00	0.18	± 0.00
Axial Length (mm)	Control (n=9)	2.95	± 0.02	3.04	± 0.02	3.09	± 0.01	3.13	± 0.01	3.18	± 0.01
	Lens-treated (n=6)	2.99	± 0.02	3.05	± 0.02	3.12	± 0.02	3.17	± 0.01	3.20	± 0.02

Table S2. *Gnat2*$^{-/-}$ ocular parameters represented as mean ± SEM.

WHITE

Ocular Parameter	Days Post-natal	28	35	42	49	56
Radius of Corneal Curvature (mm)	Control (n=7-9)	1.42 ± 0.03	1.47 ± 0.03	1.51 ± 0.03	1.52 ± 0.02	1.54 ± 0.02
	Lens-treated (n=4-6)	1.48 ± 0.01	1.49 ± 0.00	1.54 ± 0.01	1.55 ± 0.01	1.58 ± 0.01
Corneal Thickness (mm)	Control (n=9)	0.08 ± 0.00	0.08 ± 0.00	0.08 ± 0.00	0.08 ± 0.00	0.08 ± 0.00
	Lens-treated (n=5-6)	0.08 ± 0.00	0.08 ± 0.00	0.08 ± 0.00	0.08 ± 0.00	0.08 ± 0.00
Anterior Chamber Depth (mm)	Control (n=9)	0.32 ± 0.01	0.34 ± 0.00	0.35 ± 0.00	0.37 ± 0.00	0.38 ± 0.00
	Lens-treated (n=5-6)	0.32 ± 0.00	0.34 ± 0.01	0.36 ± 0.01	0.38 ± 0.01	0.39 ± 0.00
Lens Thickness (mm)	Control (n=9)	1.75 ± 0.01	1.81 ± 0.01	1.87 ± 0.01	1.92 ± 0.01	1.96 ± 0.01
	Lens-treated (n=5-6)	1.74 ± 0.01	1.82 ± 0.01	1.88 ± 0.01	1.92 ± 0.00	1.96 ± 0.01
Vitreous Chamber Depth (mm)	Control (n=9)	0.65 ± 0.01	0.64 ± 0.01	0.62 ± 0.01	0.60 ± 0.01	0.59 ± 0.01
	Lens-treated (n=5-6)	0.66 ± 0.01	0.63 ± 0.01	0.62 ± 0.01	0.61 ± 0.01	0.60 ± 0.00
Retinal Thickness (mm)	Control (n=9)	0.18 ± 0.00	0.18 ± 0.00	0.17 ± 0.00	0.18 ± 0.00	0.18 ± 0.00
	Lens-treated (n=5-6)	0.18 ± 0.00	0.18 ± 0.00	0.18 ± 0.00	0.18 ± 0.00	0.18 ± 0.00
Axial Length (mm)	Control (n=9)	2.98 ± 0.02	3.05 ± 0.02	3.10 ± 0.01	3.14 ± 0.01	3.19 ± 0.01
	Lens-treated (n=5-6)	2.98 ± 0.01	3.05 ± 0.01	3.12 ± 0.01	3.16 ± 0.01	3.21 ± 0.01

GREEN

Ocular Parameter	Days Post-natal	28	35	42	49	56
Radius of Corneal Curvature (mm)	Control (n=8-9)	1.41 ± 0.03	1.47 ± 0.02	1.50 ± 0.03	1.52 ± 0.02	1.53 ± 0.02
	Lens-treated (n=6-7)	1.45 ± 0.02	1.50 ± 0.01	1.53 ± 0.01	1.54 ± 0.01	1.57 ± 0.01
Corneal Thickness (mm)	Control (n=8-9)	0.08 ± 0.00	0.07 ± 0.00	0.08 ± 0.00	0.08 ± 0.00	0.08 ± 0.00
	Lens-treated (n=5-7)	0.08 ± 0.00	0.08 ± 0.00	0.08 ± 0.00	0.07 ± 0.00	0.08 ± 0.00
Anterior Chamber Depth (mm)	Control (n=8-9)	0.31 ± 0.01	0.34 ± 0.01	0.36 ± 0.00	0.37 ± 0.00	0.37 ± 0.00
	Lens-treated (n=5-7)	0.31 ± 0.00	0.34 ± 0.00	0.36 ± 0.00	0.38 ± 0.00	0.39 ± 0.01
Lens Thickness (mm)	Control (n=8-9)	1.74 ± 0.01	1.81 ± 0.01	1.87 ± 0.01	1.91 ± 0.01	1.95 ± 0.01
	Lens-treated (n=5-7)	1.74 ± 0.01	1.80 ± 0.01	1.87 ± 0.01	1.91 ± 0.01	1.94 ± 0.01
Vitreous Chamber Depth (mm)	Control (n=8-9)	0.66 ± 0.01	0.65 ± 0.01	0.61 ± 0.01	0.61 ± 0.01	0.59 ± 0.01
	Lens-treated (n=5-7)	0.64 ± 0.01	0.63 ± 0.00	0.62 ± 0.00	0.61 ± 0.01	0.58 ± 0.01
Retinal Thickness (mm)	Control (n=8-9)	0.18 ± 0.00	0.17 ± 0.00	0.18 ± 0.00	0.18 ± 0.00	0.17 ± 0.00
	Lens-treated (n=5-7)	0.19 ± 0.00	0.18 ± 0.00	0.18 ± 0.00	0.18 ± 0.00	0.18 ± 0.00
Axial Length (mm)	Control (n=8-9)	2.97 ± 0.02	3.04 ± 0.02	3.10 ± 0.01	3.14 ± 0.02	3.17 ± 0.01
	Lens-treated (n=5-7)	2.97 ± 0.01	3.04 ± 0.01	3.11 ± 0.01	3.15 ± 0.01	3.18 ± 0.01

VIOLET

Ocular Parameter	Days Post-natal	28	35	42	49	56
Radius of Corneal Curvature (mm)	Control (n=7-10)	1.43 ± 0.02	1.48 ± 0.02	1.51 ± 0.02	1.53 ± 0.02	1.54 ± 0.01
	Lens-treated (n=3-9)	1.49 ± 0.02	1.50 ± 0.01	1.53 ± 0.01	1.54 ± 0.01	1.57 ± 0.02
Corneal Thickness (mm)	Control (n=10)	0.08 ± 0.00	0.08 ± 0.00	0.08 ± 0.00	0.08 ± 0.00	0.08 ± 0.00
	Lens-treated (n=5-9)	0.08 ± 0.00	0.08 ± 0.00	0.07 ± 0.00	0.08 ± 0.00	0.08 ± 0.00
Anterior Chamber Depth (mm)	Control (n=10)	0.31 ± 0.00	0.34 ± 0.00	0.35 ± 0.00	0.36 ± 0.00	0.38 ± 0.00
	Lens-treated (n=5-9)	0.32 ± 0.01	0.35 ± 0.00	0.37 ± 0.00	0.38 ± 0.00	0.39 ± 0.00
Lens Thickness (mm)	Control (n=10)	1.74 ± 0.01	1.80 ± 0.01	1.87 ± 0.01	1.92 ± 0.01	1.96 ± 0.01
	Lens-treated (n=5-9)	1.75 ± 0.01	1.82 ± 0.01	1.89 ± 0.01	1.92 ± 0.01	1.96 ± 0.01
Anterior Chamber Depth (mm)	Control (n=10)	0.63 ± 0.01	0.62 ± 0.01	0.61 ± 0.01	0.59 ± 0.00	0.58 ± 0.01
	Lens-treated (n=5-9)	0.66 ± 0.01	0.62 ± 0.01	0.61 ± 0.01	0.60 ± 0.01	0.58 ± 0.00
Retinal Thickness (mm)	Control (n=10)	0.18 ± 0.00	0.18 ± 0.00	0.18 ± 0.00	0.18 ± 0.00	0.18 ± 0.00
	Lens-treated (n=5-9)	0.18 ± 0.00	0.18 ± 0.00	0.18 ± 0.00	0.18 ± 0.00	0.18 ± 0.00
Axial Length (mm)	Control (n=10)	2.95 ± 0.02	3.04 ± 0.02	3.09 ± 0.01	3.13 ± 0.01	3.18 ± 0.01
	Lens-treated (n=5-9)	2.99 ± 0.02	3.05 ± 0.02	3.12 ± 0.02	3.17 ± 0.01	3.20 ± 0.02
